# B-cell lymphoblastic lymphoma following intravenous BNT162b2 mRNA booster in a BALB/c mouse: A case report

**DOI:** 10.3389/fonc.2023.1158124

**Published:** 2023-05-01

**Authors:** Sander Eens, Manon Van Hecke, Kasper Favere, Thomas Tousseyn, Pieter-Jan Guns, Tania Roskams, Hein Heidbuchel

**Affiliations:** ^1^ Laboratory of Physiopharmacology, Genetics, Pharmacology and Physiopathology of Heart, Blood Vessels and Skeleton (GENCOR), University of Antwerp, Antwerp, Belgium; ^2^ Research Group Cardiovascular Diseases, Genetics, Pharmacology and Physiopathology of Heart, Blood Vessels and Skeleton (GENCOR), University of Antwerp, Antwerp, Belgium; ^3^ Laboratory of Translational Cell and Tissue Research, Department of Imaging and Pathology, University of Leuven, Leuven, Belgium; ^4^ Department of Cardiology, Antwerp University Hospital, Antwerp, Belgium; ^5^ Department of Internal Medicine, Ghent University, Ghent, Belgium

**Keywords:** lymphoma, B-cell, lymphoblastic, COVID-19, mRNA vaccine, BNT162b2, mouse, BALB/c

## Abstract

Unprecedented immunization campaigns have been rolled out worldwide in an attempt to contain the ongoing COVID-19 pandemic. Multiple vaccines were brought to the market, among two utilizing novel messenger ribonucleic acid technology. Despite their undisputed success in decreasing COVID-19-associated hospitalizations and mortality, various adverse events have been reported. The emergence of malignant lymphoma is one of such rare adverse events that has raised concern, although an understanding of the mechanisms potentially involved remains lacking. Herein, we present the first case of B-cell lymphoblastic lymphoma following intravenous high-dose mRNA COVID-19 vaccination (BNT162b2) in a BALB/c mouse. Two days following booster vaccination (*i.e.*, 16 days after prime), at only 14 weeks of age, our animal suffered spontaneous death with marked organomegaly and diffuse malignant infiltration of multiple extranodal organs (heart, lung, liver, kidney, spleen) by lymphoid neoplasm. Immunohistochemical examination revealed organ sections positive for CD19, terminal deoxynucleotidyl transferase, and c-MYC, compatible with a B-cell lymphoblastic lymphoma immunophenotype. Our murine case adds to previous clinical reports on malignant lymphoma development following novel mRNA COVID-19 vaccination, although a demonstration of direct causality remains difficult. Extra vigilance is required, with conscientious reporting of similar cases and a further investigation of the mechanisms of action explaining the aforementioned association.

## Introduction

Shortly after its initial outbreak in the Wuhan city of China, severe acute respiratory syndrome coronavirus 2 (SARS-CoV-2) and its associated disease, Coronavirus Disease 2019 (COVID-19), have led to a global pandemic with major socio-economic impact and millions of confirmed deaths (1, 2). Unprecedented mass immunization campaigns have been initiated worldwide in an attempt to contain the ongoing pandemic. Multiple COVID-19 vaccines, utilizing viral vector-based and novel messenger ribonucleic acid (mRNA) technology, were developed at record speed and brought to the market, with clinical trials demonstrating their efficacy and short-term safety profile before rollout (3–5). In spite of their undisputed success in decreasing COVID-19-associated hospitalizations and mortality, with over 13 billion doses administered globally, a variety of adverse events associated with COVID-19 vaccination have been reported among the general population (6, 7). The emergence of malignant lymphoma following mRNA COVID-19 vaccination is one of such rare adverse events that has raised concern, although evidence for causality and a deeper understanding of the mechanisms potentially involved are lacking (8).

In this report, we present a first case of fatal B-cell lymphoblastic lymphoma (B-LBL) diagnosed shortly following intravenous booster administration of the BNT162b2 mRNA COVID-19 vaccine in a BALB/c mouse.

## Case description

**Figure 1:**
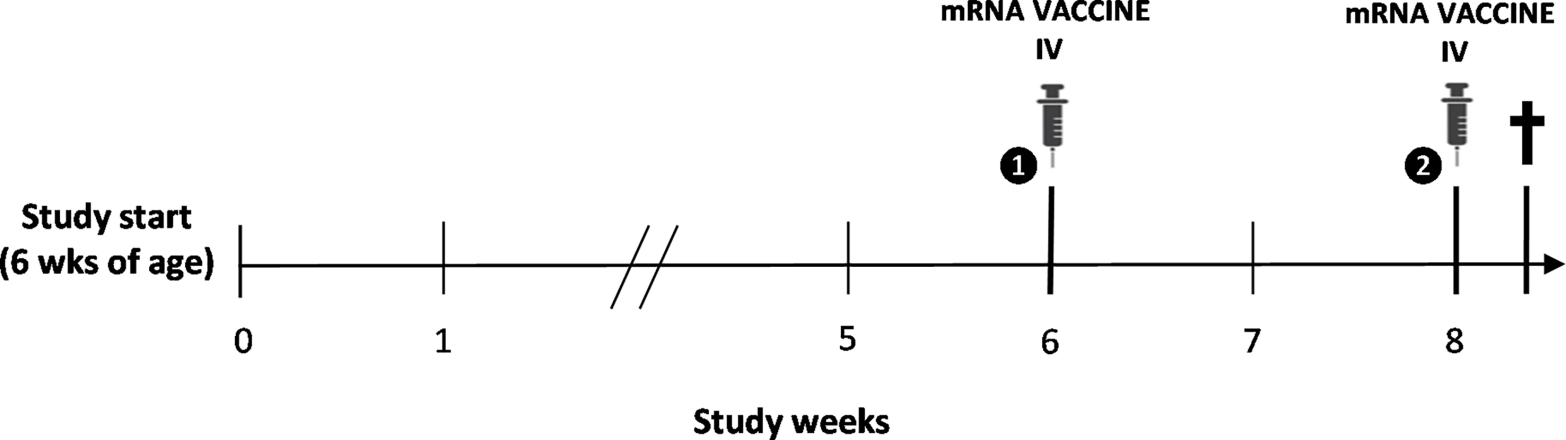
Schematic overview of the study design. Intravenous (IV) immunization with the BNT162b2 mRNA COVID-19 vaccine was performed following a two-dose regimen at 12 and 14 weeks of age, with spontaneous death of the animal two days following booster vaccination.

Five-week-old male BALB/c (substrain OlaHsd) mice were purchased from a commercial animal breeder (ENVIGO, Horst, The Netherlands) for use in an experimental study aimed to establish a mouse model of mRNA COVID-19 vaccine-induced myocarditis, as described earlier (approval nos. 2021-67 and 2022-68 (*University of Antwerp Ethical Committee*)) (see **Figure 1**) (9). To this end, one group of animals (n=14) was immunized intravenously *via* the lateral tail vein with the BNT162b2 mRNA COVID-19 vaccine (Pfizer-BioNTech) following a two-dose regimen, while another group (n=14) received normal saline injections and served as control. The second dose of BNT162b2 was administered 14 days after the first priming dose, at 12 and 14 weeks of age, respectively. Each dose contained 6 µg of BNT162b2 diluted in a total volume of 60 µl of normal saline (± 0.25 µg BNT162b2 per gram of body weight). All animals were housed in standard type III plastic cages (with a maximum of seven mice per cage) and maintained at controlled room temperature (20-24°C) and relative humidity (45-65%) under a 12-h light/dark (L/D) cycle. Animals were fed standard pelleted rodent chow and had access to water *ad libitum*. All treatments and tests were carried out during the light phase of the L/D cycle.

**Figure 2:**
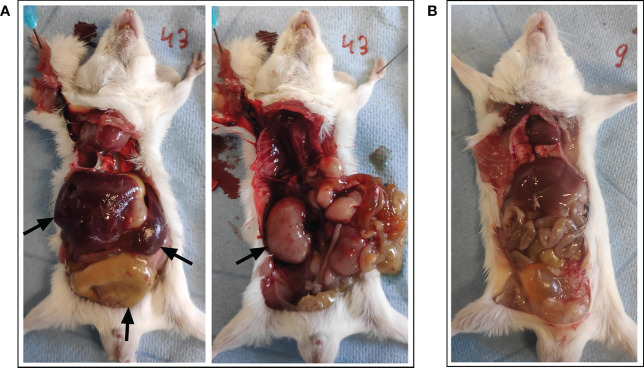
Necropsy examination of organs following spontaneous death. **(A)** A disproportional enlargement of several of the animal’s major organs was observed at necropsy, including the liver, kidneys, spleen, and intestines (*black arrows*). **(B)** Animal with normal phenotype for reference.

On the predetermined day of study termination, two days following BNT162b2 mRNA booster vaccination (i.e., 16 days after prime), one animal was found dead in its housing cage hours before planned sacrifice. There was no specific lead-up to this spontaneous death since no abnormalities were observed during daily examination for follow-up of animal welfare. Moreover, the animal was still routinely weighed in the morning prior to the sudden death without displaying externally evident clinical symptoms. At necropsy examination, a disproportional enlargement of several major thoracic and abdominal organs was observed, including the liver, kidneys, spleen, lungs, and intestines (see **Figure 2**).

### Histopathological and immunohistochemical analysis

**Figure 3:**
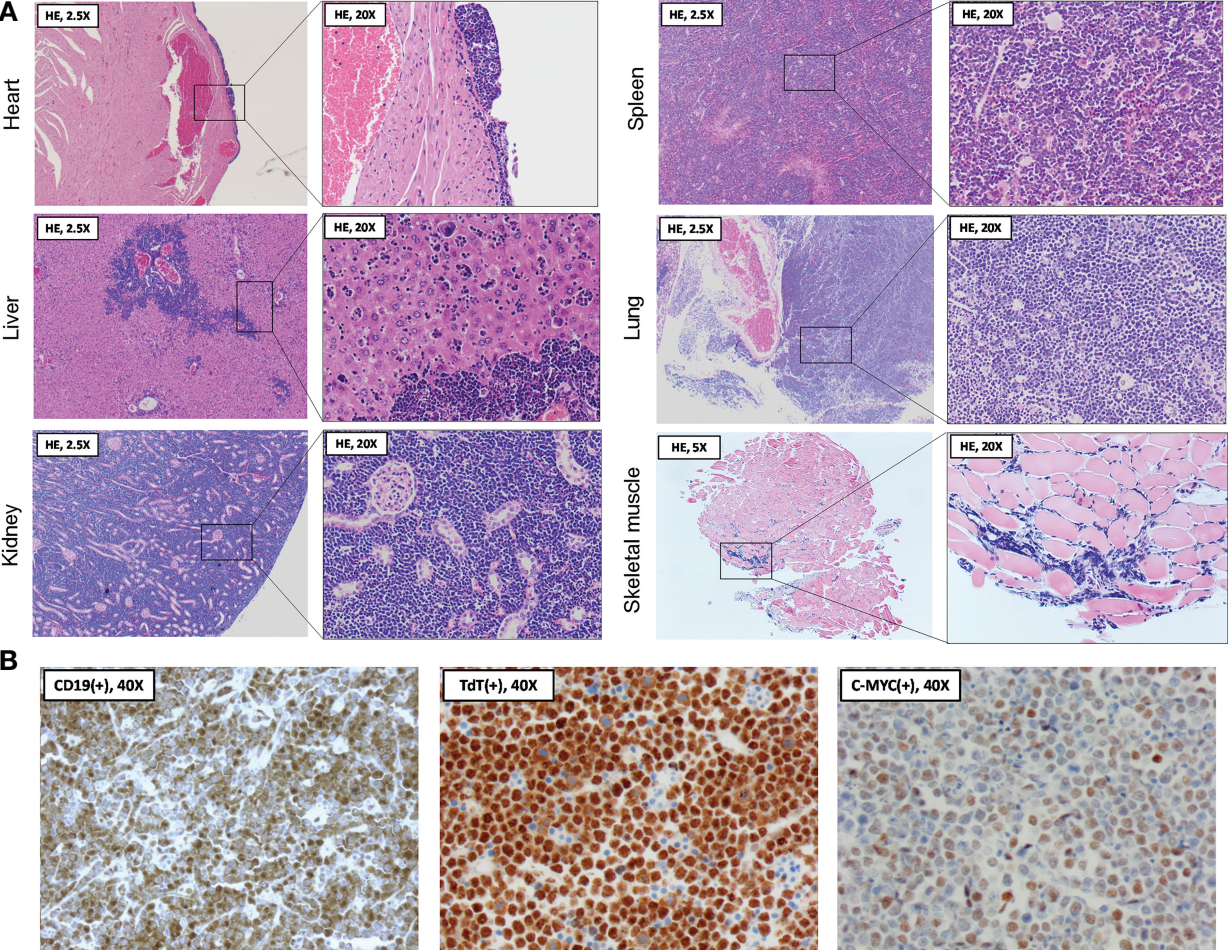
Histopathological and immunohistochemical analysis of organs following spontaneous death. **(A)** At necropsy two days following BNT162b2 booster, heart, liver, kidneys, spleen, lungs, and skeletal muscle were collected and processed for hematoxylin and eosin (HE) staining. Histopathological examination of the organ sections revealed a diffuse and extensive infiltration by medium-sized atypical cells, often completely obliterating the normal parenchyma. The skeletal muscle section showed evidence of systemic leukocytosis without parenchymal abnormalities. **(B)** Immunohistochemical findings showing staining of medium-sized atypical cells positive for B-cell marker CD19 (left panel), with further nuclear staining positive for terminal deoxynucleotidyl transferase (TdT) (middle panel) and the c-MYC oncoprotein (right panel). Stainings are compatible with a B-cell lymphoblastic lymphoma (B-LBL) immunophenotype.

Following organ dissection, tissues were formalin-fixed and paraffin-embedded, cut into 5 µm sections, and hematoxylin and eosin (HE) stained for histopathological examination (3801540BBE and 3801590BBE by Leica Biosystems, Wetzlar, Germany, see **Figure 3A**). Cardiac sections showed a normal aspect of the cardiomyocytes, with congestion of the large blood vessels and capillaries in the myocardium. At the epicardial surface, a localized, solid population of medium-sized atypical cells was present. These atypical cells showed large, polygonal nuclei with coarse chromatin, a small nucleolus, and mitotic figures. Many of the atypical cells were apoptotic with abundant presence of foamy macrophages containing debris, creating a ‘starry sky’ appearance. The epicardial focus extended slightly between the cardiomyocytes. In other regions of the epicardium, reactive changes of visceral pericardial cells were observed. The liver showed infiltration by the same population of atypical cells as described above, located in the sinusoids and surrounding the portal blood vessels. Moreover, numerous scattered megakaryocytes were present in the parenchyma, indicating extramedullary hematopoiesis. The renal interstitium was completely overgrown and distended by the same population of atypical cells as described earlier. Only few glomeruli and tubuli were recognizable in the corticomedullary region. Although they appeared intact on microscopy, their normal back-to-back arrangement was distorted due to the interstitial expansion. In the spleen, the amount of white pulp was strongly increased, with only minimal red pulp remaining. Again, the white pulp was completely infiltrated by the same population of atypical cells as described earlier. Similar to the liver, a large number of scattered megakaryocytes were observed in the parenchyma. In the sections of the lungs, only a limited amount of pulmonary parenchyma was recognizable. Most of the parenchyma was overgrown by the same population of atypical cells as described earlier, originating from the perivascular space. The extensor digitorum longus (EDL) muscle was examined to investigate the potential involvement of skeletal muscle. Although the capillaries contained an increased number of inflammatory cells, suggesting systemic leukocytosis, no further histopathological abnormalities were observed. Unfortunately, the intestines, pancreas, and lymph nodes were not dissected during necropsy, nor was blood sampling feasible given that the circulation had already stopped.

The above histopathological observations suggested extensive, systemic infiltration of the organs by a malignant lymphoid neoplasm, morphologically most suggestive of a Burkitt lymphoma (BL) or B-cell lymphoblastic lymphoma (B-LBL). For confirmation and further differentiation of the lymphoma diagnosis, immunohistochemistry (IHC) was performed using commercially available antibodies against CD19, terminal deoxynucleotidyl transferase (TdT) and c-MYC (see **Figure 3B**). Anti-CD19 staining (diluted 1:100, catalog no. 90176, Cell Signaling, Danvers, US) confirmed the B-cell origin of the neoplastic cells. Moreover, staining with anti-TdT (diluted 1:200, catalog no. ab85148, Abcam, Cambridge, UK) showed nuclear expression of TdT in the neoplastic cells. TdT is a marker of immaturity expressed in nearly all (i.e., 90 - 95%) LBL cases but lacks in BL (10). Lastly, anti-c-MYC staining (diluted 1:200, catalog no. ab32072, Abcam, Cambridge, UK) demonstrated increased c-MYC expression in the neoplastic cell nuclei, potentially indicating a c-MYC rearrangement as one of the lymphoma driver mutations. Such rearrangement is associated with several hematologic malignancies, including both BL and B-LBL (11). However, in the present case, BL is unlikely due to the TdT expression of the neoplastic cells, strengthening the B-LBL diagnosis.

No SARS-CoV-2 spike protein was observed in any of the organs studied (data not shown, using a rabbit polyclonal anti-SARS-CoV-2 spike protein antibody (diluted 1:200, catalog no. NB100-56578, Bio-Techne Ltd., Minneapolis, US)). Lung- and heart tissue of a male SCID mouse that underwent a confirmed SARS-CoV-2 infection served as positive control for this staining.

### Body weight evolution

**Figure 4:**
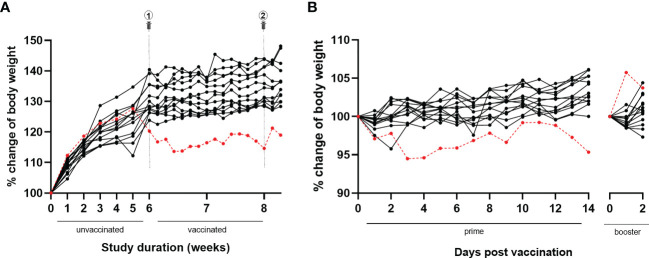
Body weight evolution throughout the study and following BNT162b2 prime and booster. **(A)** During the full study course, the animal of this case report (*red dotted line*) was weighed weekly until primary vaccination and daily afterwards. Over the first five study weeks, the animal’s body weight gradually increased reaching peak body weight. Afterwards, a drop in body weight was observed, without recovery. In contrast, all other animals belonging to the same experimental group (*black lines*) showed a progressive increase of body weight over time. **(B)** Similar to the other animals belonging to the same experimental group (*black lines*), the animal of this case report (*red dotted line*) showed a slight decrease in body weight following primary immunization. In contrast with the other animals, a remarkable 5.7% increase in body weight was observed following booster immunization.

During the study course, animals were weighed weekly for six weeks until the day of first immunization and thereafter daily. At the first weighing after a one-week acclimatization period in our animal facility, the animal showed a body weight of 21.4 g (group mean ± SD: 20.3 ± 1.2 g). Over the next study weeks, the animal’s body weight gradually increased reaching peak body weight at week five (i.e., one week prior to the first immunization) (see **Figure 4A**). Onwards, however, an unexpected and remarkable drop in body weight was observed over the next 10 days, in contrast to the other animals of the experimental group, which continued to slowly gain body weight with aging. The body weight of the animal did not recover over the remaining course of the study. The response to vaccination was similar to the other animals for the primary vaccination (slight decrease in body weight), in contrast to the booster vaccination (5.7% body weight increase in the animal versus slight decrease in most of the other animals) (see **Figure 4B**).

## Discussion

Two days following booster vaccination, the animal suffered spontaneous death with diffuse malignant infiltration of multiple extranodal organs at only 14 weeks of age. Although a causal relationship between the SARS-CoV-2 mRNA vaccine and B-LBL observed in the present case cannot be unequivocally established and may represent coincidence, the temporal sequence of events suggests its involvement in this rare hematologic malignancy. With the exception of this case, no other animals experienced any adverse events post mRNA COVID-19 vaccination in our study.

To the best of our knowledge, no clinical nor experimental data exist on the development of B-LBL following COVID-19 vaccination. LBL is an aggressive neoplasm arising from lymphoblasts of either B- or T-cell origin, accounting for approximately 8% of all human lymphoid malignancies (12–14). B-LBL is the uncommon form (i.e., < 10% of all LBLs) and has a slight male predominance, usually involving the lymph nodes as well as extranodal sites, including the skin, soft tissue, and bone (15, 16).

Clinical cases with the diagnosis of other B-cell lymphoma subtypes, including diffuse large B-cell lymphoma (DLBCL) and marginal zone lymphoma (MZL), have been reported, although most often only temporarily linked with COVID-19 vaccination since a pre-existing lymphoma could not be excluded (17–19). Notably, the reported cases occurred in the context of immunization with the BNT162b2 mRNA vaccine. Nonetheless, remission and Epstein-Barr virus (EBV) reactivation of B-cell lymphoma have also been reported following the ChAdOx1 nCoV-19 viral vector vaccine (20, 21). Individual cases of development, progression, exacerbation, recurrence, and spontaneous regression of T cell lymphoma have also been described for both the mRNA-based as well as viral vector-based SARS-CoV-2 vaccines (8, 18, 22–27). Interestingly, the occurrence of malignant lymphoma post vaccination is not limited to the novel SARS-CoV-2 vaccines. Development of MZL resulting from the antigenic stimulus of influenza vaccine components has been described earlier (28). This finding was further strengthened by a population-based study reporting a positive association between the risk of non-Hodgkin lymphoma (NHL) and receiving influenza vaccination, involving DLBCL and follicular lymphoma (FL) (29).

The mRNA SARS-CoV-2 vaccines are reported to elicit a robust adaptive immune response through the potent stimulation of antigen-specific CD4^+^/CD8^+^ T-cells and selective generation of high neutralizing antibody titers against the SARS-CoV-2 spike protein (30). SARS-CoV-2-specific germinal center B-cell and plasmablast responses were shown to persist for at least 15 weeks after the first immunization in BNT162b2-vaccinated individuals (31). Although deeper mechanistic insights remain elusive, it has been suggested that the continuous stimulation of T- and B-cells, not only by SARS-CoV-2 itself but also through mRNA SARS-CoV-2 vaccination, may trigger autoimmunity or other aberrant inflammatory responses, thereby resulting in malignant lymphoma development or progression of pre-existing lymphoma (19, 32–34).

Despite the suggested involvement of the mRNA COVID-19 vaccine in this hematologic malignancy, spontaneous development and progression of the B-LBL in our animal is to be considered. Data on the occurrence of spontaneous lymphoma in laboratory mice are scarce. In a study of female BALB/c mice, B-LBLs were reported to appear primarily in old (i.e., mean age > 20 months) but not young mice (35). Moreover, the ENVIGO BALB/c model information sheet reports a zero incidence of lymphatic leukemia and a low gross tumor incidence in males (36, 37). Even in the case of a B-LBL that developed spontaneously, the SARS-CoV-2 mRNA vaccination might have had an accelerating effect on its magnitude and/or speed of progression. For example, a human case of angioimmunoblastic T-cell lymphoma with rapid progression after BNT162b2 mRNA booster vaccination has been described recently (22). Lastly, it remains unclear if, and to what extent, the animal’s body weight throughout the course of the study, including the unexpected weight drop starting one week prior to the first immunization, can be linked to the moment of lymphoma development or its stage of progression.

It should be noted that various factors in our experimental study might limit the clinical translatability. First, the BNT162b2 mRNA vaccine was administered intravenously and not *via* the designed intramuscular route of delivery. Intramuscular vaccination has been described to initiate an adaptive immune response in the lymph nodes draining the injection site, whereas little is known about the effects of direct entrance and consequent distribution of lipid nanoparticle (LNP)-encapsulated mRNA in the systemic circulation (30, 38). Nonetheless, occasional blood aspiration following inadvertent intravenous injection of SARS-CoV-2 vaccines has been reported earlier (38, 39). Second, with each immunization, the animal received a disproportionately larger dose of BNT162b2 per gram of body weight than would be the case in human use (i.e., a normal dose contains 30 µg BNT162b2 mRNA). Correspondingly, a much greater SARS-CoV-2 mRNA vaccine-specific immune response may have been elicited by each of the two BNT162b2 mRNA vaccinations.

Given the paucity of data on the long-term safety of the SARS-CoV-2 mRNA vaccines, it is vital that clinicians and scientists report any adverse event to establish potential correlations. Our case adds to previous clinical reports on malignant lymphoma development following novel SARS-CoV-2 mRNA vaccination. Interestingly, we are the first to report a B-LBL subtype, with its occurrence in mouse allowing a detailed histopathological and immunohistochemical examination of the different organs involved. Although strong evidence proving or refuting a causal relationship between SARS-CoV-2 mRNA vaccination and lymphoma development or progression is lacking, vigilance is required, with conscientious reporting of similar cases and a further investigation of the mechanisms of action that could explain the aforementioned association.

## Data availability statement

The original contributions presented in the study are included in the article/supplementary material. Further inquiries can be directed to the corresponding author.

## Ethics statement

The animal study was reviewed and approved by University of Antwerp Ethical Committee.

## Author contributions

SE performed the experiments and drafted the manuscript. MH, TT, and TR performed the histopathological evaluation. All co-authors critically reviewed and edited the manuscript. All authors contributed to the article and approved the submitted version.
