# Addendum: B-cell lymphoblastic lymphoma following intravenous BNT162b2 mRNA booster in a BALB/c mouse: a case report

**DOI:** 10.3389/fonc.2023.1267904

**Published:** 2023-10-20

**Authors:** Sander Eens, Manon Van Hecke, Kasper Favere, Thomas Tousseyn, Pieter-Jan Guns, Tania Roskams, Hein Heidbuchel

**Affiliations:** ^1^ Laboratory of Physiopharmacology, Genetics, Pharmacology and Physiopathology of Heart, Blood Vessels and Skeleton (GENCOR), University of Antwerp, Antwerp, Belgium; ^2^ Research Group Cardiovascular Diseases, Genetics, Pharmacology and Physiopathology of Heart, Blood Vessels and Skeleton (GENCOR), University of Antwerp, Antwerp, Belgium; ^3^ Laboratory of Translational Cell and Tissue Research, Department of Imaging and Pathology, University of Leuven, Leuven, Belgium; ^4^ Department of Cardiology, Antwerp University Hospital, Antwerp, Belgium; ^5^ Department of Internal Medicine, Ghent University, Ghent, Belgium

**Keywords:** lymphoma, B-cell, lymphoblastic, COVID-19, mRNA vaccine, BNT162b2, mouse, BALB/c

Our research group published a case report in the Journal in May 2023 (DOI: 10.3389/fonc.2023.1158124) describing the unexpected spontaneous death of a BALB/c mouse two days after receiving the second dose of the Pfizer/BioNTech mRNA vaccine (1). Upon necropsy, the animal exhibited marked organomegaly with histopathologic examination revealing diffuse infiltration of multiple extranodal organs by a malignant lymphoid neoplasm. Further immunohistochemical analyses ultimately led to the diagnosis of a B-cell lymphoblastic lymphoma. Considering previously published case reports regarding a potential association between mRNA COVID-19 vaccination and malignant lymphoma development or progression, although often only temporally, our research group made the conscientious decision to report this remarkable case (2–7). In our case report, we explicitly indicated the lacking evidence for causality between mRNA vaccination and the observed lymphoma.

In the months following publication, we noticed that our case report has gained significant public attention, particularly on social media platforms. More specifically, our case report has been largely misinterpreted and used as a study providing evidence that mRNA COVID-19 vaccination can trigger a phenomenon which has non-scientifically been referred to as “turbo cancer”. First of all, we wish to unequivocally disassociate ourselves from this term. In our case report, there is not a single reference to a condition called “turbo cancer”, nor do we recognize it as a legitimate medical term. Secondly, we would like to point out that case reports are descriptive and explorative in nature, presenting unexpected medical findings in an unbiased manner. Unlike experimental studies, they do not test correlation between variables, nor are they used to demonstrate causality. Notably, at the moment of writing this correspondence, we have injected over 70 BALB/c mice with the BNT162b2 mRNA vaccine (either single- or double dosed). Excluding the published case report, none of these other vaccinated animals developed hematologic malignancies of any type.

As explicitly stated in our case description, the spontaneously deceased animal was part of a larger study in which our group aimed to replicate a previously described murine model of mRNA COVID-19 vaccine-associated myocarditis (8). In this context, our experimental design, including the mouse strain, disproportionally high dosing, intravenous administration route, and timing of sacrifice, was a full-on replication of this previously published study. Our primary study’s objective did not involve investigating the effect of mRNA COVID-19 vaccination on adverse events other than myocarditis. Moreover, the initial publication by Li et al. did also not report any hematological malignancy.

Moreover, we highlighted multiple times in our case report that a causal relationship between the mRNA COVID-19 vaccine and the observed malignancy could not be established. As for all the referenced case reports, we have specified that there might only be a temporal association and that a pre-existing lymphoma could not be excluded. We considered spontaneous development of the lymphoma, which has previously been reported in this particular mouse strain (9). Against the background of such spontaneous development, however, we took into consideration the possibility that our vaccination protocol might have had an accelerating effect on the progression of the lymphomatous lesion, as Goldman et al. had suggested earlier (6).

Regarding the body weight evolution of the affected mouse in our study (Figure 4A of our case report), we wrote: “*Lastly, it remains unclear if, and to what extent, the animal’s body weight throughout the course of the study, including the unexpected weight drop starting one week prior to the first immunization, can be linked to the moment of lymphoma development or its stage of progression*”. It is indeed plausible that the sudden drop in body weight observed in our case from week 5 to 6 prior to the first vaccine administration relates to a pre-existing lymphoma. Nevertheless, we should note that previous experiments have taught us that weekly body weight fluctuations in mice may occur, without underlying health issues or pathology. Moreover, as clearly can be seen in the same figure, another mouse of our study showed a similar drop in body weight prior to the first vaccination (*i.e.*, from week 4 to 5) without demonstration of grossly visible or histopathological changes at sacrifice.

In conclusion, the novel COVID-19 vaccines have demonstrated an exceptional benefit-risk ratio in the fight against the pandemic and manifestations of severe adverse reactions following COVID-19 vaccination are rare. Our case report in not any way counters that overwhelming benefit-risk profile. Nonetheless, we firmly believe that is vital for scientists and clinicians to remasin vigilant and report any potential (extremely rare) adverse event which may help in establishing future correlations.
